# The Role of Positron Emission Tomography in Advancing the Understanding of the Pathogenesis of Heart and Vascular Diseases

**DOI:** 10.3390/diagnostics13101791

**Published:** 2023-05-18

**Authors:** Anna Blach, Jacek Kwiecinski

**Affiliations:** 1 Department of Cardiology and Structural Heart Diseases, Medical University of Silesia, 40-055 Katowice, Poland; 2 Nuclear Medicine Department, Voxel Diagnostic Center, 40-514 Katowice, Poland; 3 Department of Interventional Cardiology and Angiology, Institute of Cardiology, 04-628 Warsaw, Poland

**Keywords:** positron emission tomography, myocardial viability, infective endocarditis, coronary artery disease, ^18^F-sodium fluoride

## Abstract

Cardiovascular disease remains the leading cause of morbidity and mortality worldwide. For developing new therapies, a better understanding of the underlying pathology is required. Historically, such insights have been primarily derived from pathological studies. In the 21st century, thanks to the advent of cardiovascular positron emission tomography (PET), which depicts the presence and activity of pathophysiological processes, it is now feasible to assess disease activity in vivo. By targeting distinct biological pathways, PET elucidates the activity of the processes which drive disease progression, adverse outcomes or, on the contrary, those that can be considered as a healing response. Given the insights provided by PET, this non-invasive imaging technology lends itself to the development of new therapies, providing a hope for the emergence of strategies that could have a profound impact on patient outcomes. In this narrative review, we discuss recent advances in cardiovascular PET imaging which have greatly advanced our understanding of atherosclerosis, ischemia, infection, adverse myocardial remodeling and degenerative valvular heart disease.

## 1. Introduction

Positron emission tomography (PET) is a non-invasive, functional imaging test utilizing ionizing radiation, the source of which is a radioactive isotope (radionuclide) administered to the patient. By measuring the radioactivity in the examined organs, PET enables tracking dynamic biological processes in vivo on three-dimensional images. The principle of cardiac PET imaging is based on intravenous administration of a radionuclide-tagged tracer molecule (radiotracer) and subsequent registration of the radiation emitted by it by the detector in which the patient is placed.

## 2. Physics

A wide range of radionuclides for PET molecular imaging of the heart are available: ^18^F, ^13^N, ^82^Rb, ^15^O and ^68^Ga; as unstable radioactive elements, they exhibit spontaneous decay. The unstable state is not a feature characteristic for most naturally existing atoms—but it can be artificially achieved with the transmutation of elements in an accelerator. To restore a stable state, a radionuclide undergoes a transformation of its core via positron emission, according to β+ (positron) decay:

$$p\to n+\beta^{++v}$$

where p—proton, n—neutron, v—neutrino and $\beta^{+}$—positron (beta particle, positively charged electron).

**Figure 1:**
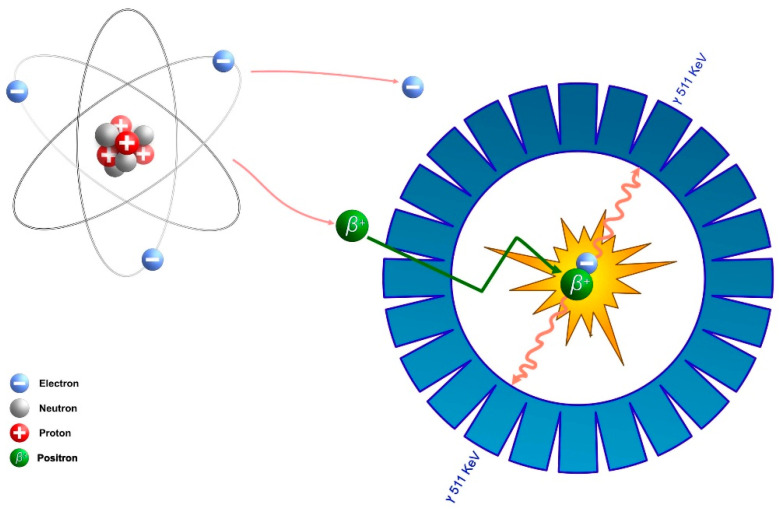
Schematic representation of the basic principles governing PET. A positive-beta decay: interaction of positron with electron followed by an annihilation process with the release of two 511 keV gamma photons detected by the PET scanner detector ring.

A positron is a positive-charged anti-matter to an electron. Emitted from the unstable nucleus, it travels within the surrounding tissue, gradually losing its kinetic energy through interactions with nearby bond-electrons to finally encounter a loose electron to annihilate with, resulting in the conversion of their masses into the energy of two photons ejected simultaneously in two opposite directions, each carrying the energy of 511 KeV [1,2], Figure 1.

Before reaching the PET scanner detection ring, photons interact with other charged particles, which leads to Compton scattering, i.e., loss of energy and change in direction, reducing the photon flux. The likelihood and degree of absorption or scattering (attenuation) is proportional to tissue density and detector distance, negatively influencing the sensitivity and spatio-temporal resolution. Hence, to obtain high-quality images and accurate quantitation of tracer uptake, attenuation correction must be applied. Compared with past generations of stand-alone PET scanners, nowadays PET–CT scanners allow for CT attenuation and scatter correction, resolving the two most prominent limitations of the first generation of PET. Typically acquired in parallel to the PET emission scan, CT or MR can additionally be reviewed for anatomical reference, including diagnostic information such as the presence of coronary calcium and/or extra-cardiac morphological findings within a single imaging session [3].

In cardiovascular imaging, PET can be leveraged for viability, perfusion, infiltrative disease and, more recently, for atherosclerotic plaque and valvular degeneration assessments. The two most widely employed applications of cardiovascular PET are viability and perfusion (flow) imaging. The former relies on identifying those cardiomyocytes that are alive, defined by the presence of cellular, metabolic function; the latter refers to the evaluation of blood supply to the myocardium, which can be hampered due to flow-limiting stenoses within the epicardial coronary vessels and/or the microvasculature.

## 3. Myocardial Perfusion and Flow Imaging

Myocardial perfusion imaging (MPI) with radionuclide PET is one of the non-invasive imaging methods that can provide rapid and accurate information about the extent of ischemia. The gold standard for assessing perfusion is the use of O^15^-labeled water, a freely diffusible tracer with nearly 100% first-pass extraction from the blood [4,5]. A major limitation of this tracer is its short half-life of about 2 min, which results in the need for a cyclotron within the PET scanner neighborhood so that it can be produced and administered to the patient in a very short timeframe. Consequently, non-diffusion tracers such as N^13^-ammonia (^13^NH_3_) and 82-rubidium chloride (^82^Rb), which either have a longer half-life (^13^NH_3_) or are produced in a generator in the PET scanner room (^82^Rb), are more widely employed for MPI (Table 1 and Table 2) [6,7].

The obtained resting and stress perfusion images are compared, determining the presence, location and size of perfusion defects qualitatively and semi-quantitatively. By semi-quantitatively estimating the severity of the defect, one relates the degree of tracer uptake in each segment to that in which the uptake is greatest—thus obtaining relative perfusion data. When perfusion in the reference area is impaired, these data are inadequate for the actual flow rate, leading to false-negative findings—an issue that is particularly relevant in patients with multivessel coronary artery disease, as well as left main disease [8,9]. As shown in a recent study, this situation occurs in as many as 4.5% of symptomatic patients with multivessel disease [10]. Another important cohort comprises patients with normal coronary arteries in whom microvascular dysfunction is suspected: especially women, diabetic patients and patients with chronic kidney disease [6]. These obstacles have been effectively eliminated with the introduction of dynamic flow acquisitions with quantitative assessments of global and regional myocardial blood flow (MBF) and myocardial flow reserve (MFR), which is the ratio of maximal flow in a hyperemic state to myocardial blood flow at rest.

Since the first studies evaluating coronary reserve in relation to the degree of vascular stenosis were published almost 50 years ago [11], the clinical relevance of flow reserve has been widely recognized. While nowadays assessments of flow reserve are primarily performed invasively during coronary catheterization, such assessments can be conducted using PET. A great advantage of PET MBF measurements is not simply that they are noninvasive, but more importantly that they analyze not only the relative data resulting from flow through a portion of a single vessel [12], but they demonstrate the absolute values of coronary flow through the entire thickness of the tissues involved, with no additional radiation and exposures as low as 0.5 mSv [13,14,15]. The method has been shown to be highly reproducible [16], and maintains a high prognostic value independent of the body mass index (BMI) [17,18]. According to COVADIS, impaired coronary flow reserve with cutoff values ≤2.0 and impaired hyperemic MBF has been recognized as one of the diagnostic criteria for microvascular angina [19].

## 4. Viability

There is a complex relationship between myocardial perfusion, mechanical function and metabolism. Numerous studies have evaluated the accuracy of viability imaging for prospective identification of patients with ischemic cardiomyopathy and potentially reversible left ventricular (LV) dysfunction who can benefit from future revascularization [19,20,21,22,23]. With respect to metabolic or contractile reserve, LV impairment may have two causes: (a) irremediable necrosis equal to scarring and (b) different but overlapping reversible stunning and hibernation. The most established nuclear medicine technique to image heart metabolism utilizes ^18^F-fluorodeoxyglucose (^18^FDG)-PET, and since 1986 it continues to serve as a “gold standard” to differentiate a scar from viable hibernated myocardium [24].

**Figure 2:**
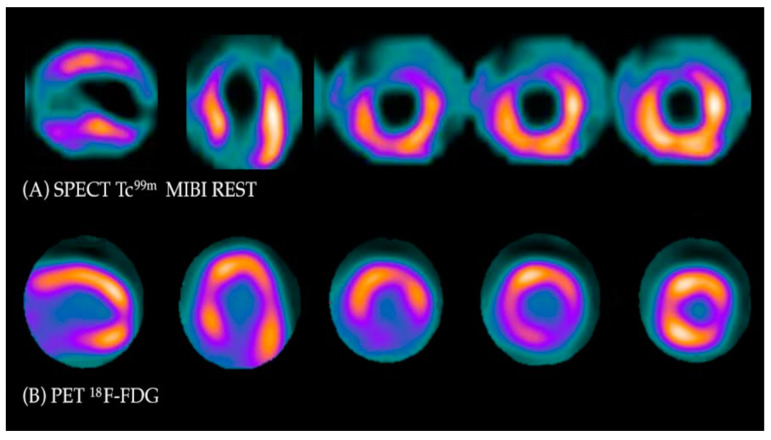
Myocardial viability imaging: Mismatch pattern, viable hibernated myocardium. (**A**) Myocardial perfusion imaging with MIBI-Tc^99m^ SPECT: heart scans showing lack of tracer uptake in the apical region and apical segments of the anterior wall (left anterior descending coronary artery territory). (**B**) ^18^FDG viability PET. ^18^FDG uptake is visible in the area of the perfusion deficit, ruling out scarring and confirming viability in this region.

**Figure 3:**
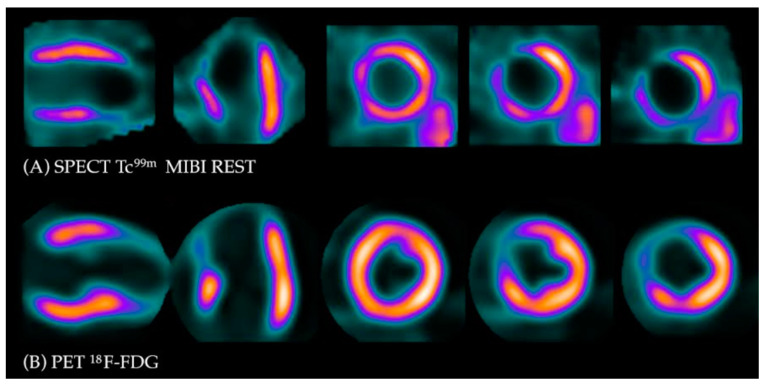
Myocardial viability imaging: Match pattern, non-viable scarring. (**A**) Myocardial perfusion imaging with MIBI-Tc^99m^ SPECT: heart scans showing lack of tracer uptake in the apical region of the left ventricle (LAD territory). (**B**) ^18^FDG viability PET. ^18^FDG uptake is absent in the area of the perfusion defect, confirming myocardial scarring in this region.

Under normal conditions, myocytes as the “omnivorous” cells use free fatty acids (FFAs) as a preferable source of energy via the highly energetic, oxygen-dependent process of beta-oxidation. Prolonged ischemia is a status when the main source of energy is switched to glucose derived from anaerobic glycolysis, and in consequence leads to a substantial increase in glucose utilization [25]. This phenomenon can be depicted with PET. As a glucose analog, ^18^FDG is absorbed by cardiac myocytes, becoming a surrogate marker of myocardial glucose uptake. ^18^FDG is actively transported into the cells by GLUT-1 and four glucose transporters in the same way as a “normal” glucose molecule. As ^18^FDG-6-phosphate cannot be transformed back to ^18^FDG, it is eventually trapped inside the cell, providing an opportunity for a non-invasive assessment of glucose metabolism with PET [25]. ^18^FDG PET viability evaluation involves a combination of rest MPI (either PET or single photon emission computed tomography SPECT) and ^18^FDG metabolic imaging in order to determine one of three patterns of perfusion vs. viability: (1) normal conditions—preserved myocardial perfusion and viability. (2) Viable hibernation, a mismatch—reduced perfusion and preserved viability. (3) Non-viable scar, a match—reduced MPI and viability (Figure 2 and Figure 3) [26,27].

The current societal guidelines by AHA/ACC/HFSA in 2022 [28] and ESC in 2021 [29] state with similar class II recommendation that non-invasive stress imaging, including PET, may be considered for the assessment of myocardial ischemia and viability in patients with CAD who are considered suitable for coronary revascularization.

These recommendations are based on landmark clinical trials. The first broad meta-analysis of 24 studies by Allman et al. in 2002, which included 3088 patients [30], explored the potential changes in the outcomes of patients with established coronary artery disease and LV dysfunction and demonstrated a strong association between myocardial viability on noninvasive testing and improved survival after revascularization [30]. No benefit was associated with revascularization without confirmed viability, irrespectively of the imaging modality used. This notion was not confirmed in the initial analysis of the STICH trial, where viability assessed with less advanced techniques in patients referred to surgical revascularization did not improve outcomes [31]; however, over longer follow-up, there was an improvement in all-cause and cardiovascular mortality with viability-guided surgical revascularization [32]. The rational for selecting PET for viability imaging was confirmed by another post hoc analysis. In the PARR-2 study, a significant reduction in cardiac events was observed in patients with ^18^FDG-PET-assisted management compared with patients who received optimal medical treatment in a center with easy access to ^18^FDG and integration with experienced clinical teams [33]. More recently, the REVIVED-BCIs2 prospective randomized trial did not support the hypothesis that percutaneous revascularization in combination with optimal medical treatment may improve event-free survival in patients with ischemic cardiomyopathy and viable myocardium compared with a strategy of medical treatment alone, even in patients with reduced ejection fraction [34,35]. Considering the aforementioned studies, the role of myocardial viability in guiding revascularization remains controversial. Balancing procedural risks and expected benefit from revascularization is still a key question in patients with ischemic heart failure—therefore, it seems that ^18^FDG PET myocardial viability testing is a helpful tool when it is carefully matched with the patient’s profile [36], particularly in high surgical risk patients, elderly individuals with severe LV impairment and patients with a history of prior surgical revascularization and/or complex comorbidities [36]. Additionally, patients with advanced coronary artery disease referred for high-risk revascularization of chronically occluded arteries also appear to be among those who could derive the greatest benefit from PET imaging [37,38]. Indeed, it has been previously shown that viability testing with ^18^FDG PET–CT or PET–MR in patients with CTO can identify those who shall show functional improvement following revascularization [39,40].

## 5. Multiparametric Myocardial Perfusion Imaging

Given that PET MPI provides a wealth of imaging information including perfusion, absolute blood flow and function, it can be challenging to optimally integrate these data at the point of care. The complex interplay of various perfusion, flow and functional imaging estimates from PET has therefore been extensively studied. Initial analyses applied fixed thresholds for perfusion and flow, demonstrating that the inclusion of both provides improved risk stratification [41,42]. More recently, in an attempt to combine this information in a single variable, Gould et al. established that the coronary flow capacity can identify patients at a high risk of cardiovascular events [43].

In an attempt to address this clinical need, Singh et al. employed artificial intelligence for the optimal integration of perfusion, flow and function [44]. Utilizing over 4000 PET datasets, the authors showed that an explainable deep learning model which combines multiparametric imaging data outperforms flow or perfusion when these are considered in isolation for mortality prediction. This state-of-the-art deep learning model operated directly on polar maps without the need for derivation and selection of quantitative measurements, streamlining the analysis. Importantly, the study also addressed the need for explainable artificial intelligence. By highlighting regions contributing to the deep learning score on polar maps and ranking the relative contribution from different inputs for a specific patient, it facilitates the adoption of deep learning, as it improves confidence in the results and overcomes the perception of artificial intelligence as a “black box” [45].

## 6. Infective Endocarditis

Among various applications of PET–CT, it has emerged as a powerful diagnostic tool in the setting of a suspected cardiovascular infection [46,47,48]. Infective endocarditis (IE) is an infrequent, life-threatening condition with mortality up to 40% [49,50]. Its frequency varies by gender and predisposing factors, with a steady increase in prevalence over the past two decades in high-income countries [49,51]. Paradoxically, advances in modern medicine, improved healthcare and expenditure do not appear to be a viable solution to the problem. The most probable cause of the increasing prevalence of IE is the growing population of elderly, high-risk, multi-morbid patients who receive implantable devices containing artificial materials posing a risk for IE, e.g., vascular catheters, grafts, prosthetic heart valves, occluders, cardiac implantable electronic devices (CDIEs) and left ventricle assist devices (LVADs). The predominant IE etiology is Staphylococcus aureus on native heart valves and coagulase-negative staphylococci on artificial implants, followed by viridans streptococci [52,53].

In normal conditions, the intact endocardium is resistant to bacterial colonization, although when micro-injured, it becomes susceptible to bacterial colonization [54]. Recent studies have demonstrated that ^18^FDG PET is useful for detecting infections associated not only with native valves and arteries, but also with implantable devices and grafts, with a sensitivity of 93% for prosthetic valve endocarditis (PVE) and poorer, only 22%, for native valve endocarditis [55,56]. Accurate diagnosis is frequently difficult due to limitations in first-line imaging with echocardiography and/or CT alone. The most important aspects of the treatment of infective endocarditis are early diagnosis, identification of the microorganism, its drug susceptibility, early initiation of antibiotic therapy and surgical intervention, if needed.

The mechanism of ^18^FDG uptake in infectious and inflammatory foci is based on the increased glycolytic activity in the state of tissue hypoxia [57]. It was shown that activated macrophages, neutrophil granulocytes and CD4+ leukocytes show upregulation in GLUT-1 and GLUT-3 receptors [58,59,60]. Moreover, hyperemia and increased vessel wall permeability lead to an increase in glucose influx into the cytoplasm. Many reports suggest that ^18^FDG uptake reaches a maximum during the subacute phase of infection and then gradually decreases in the chronic phase, which explains why the sensitivity of ^18^FDG PET is highest in the acute phase of infection [61,62]. Based on the literature, there are two primary indications for ^18^FDG PET imaging in individuals with suspected IE: (1) to identify and confirm that IE is localized within the heart and/or the aorta, particularly in patients with inconclusive or negative results on initial first-line imaging, and (2) to diagnose the extent of infection by depicting the frequently underdiagnosed silent remote infectious emboli.

The primary advantage of employing this technique is the decrease in IE misdiagnoses. 

^18^FDG PET–CT perivalvular uptake has been embodied in the 2015 ESC infective endocarditis guidelines in prosthetic valve endocarditis as a major criterion and extracardiac uptake as a minor criterion for both prosthetic and native valve endocarditis patients [47].

Despite the fact that native valve endocarditis represents more than half of all IE cases, the data regarding this condition remain limited [63]. In a meta-analysis including 351 cases of suspected native valve endocarditis, Kamani et al. showed a poor pooled sensitivity (36.3%) but an excellent pooled specificity (99.1%) of ^18^FDG PET–CT [64]. These findings were followed by a prospective study from Philip et al. [65] and have been consistent with prior studies [66,67], confirming altogether low sensitivity but excellent almost 100% specificity of ^18^FDG PET imaging in the diagnosis of suspected IE. The reason for poor sensitivity is complex and involves (a) small vegetation size, typically < 10 mm, (b) insufficient temporal resolution necessary to depict the rapid movement of valve leaves and (c) the inflammatory response, which is less prominent in native valve than in prosthetic valve endocarditis, with more fibrotic tissue in comparison to a larger number of active polymorphonuclear cells, respectively. Despite these limitations, the authors reported high usefulness of ^18^FDG PET in about 30% of patients with native valve endocarditis, as it allows the diagnosis of a peripheral embolism or mycotic aneurysm, thus indisputably improving the sensitivity without decreasing specificity. Nevertheless, due to its low sensitivity, negative intracardiac ^18^FDG PET–CT findings cannot, however, be used to exclude the presence of native valve endocarditis.

The authors have also proposed that the frequently observed elevated diffuse ^18^FDG splenic uptake may be considered as a possible new minor diagnostic criterion for native valve endocarditis [65]. The TEPvENDO prospective study by Duval et al. [68] provides further data supporting the use of ^18^FDG PET in patients with suspected IE. The diagnostic and patient management modifications induced by systematic whole body ^18^FDG PET–CT analysis improved IE diagnosis in patients with both prosthetic and native valves (up to one patient out of five) and modified classification and/or management in 40% of patients—which indicates that a sizable proportion of patients with both native and prosthetic valve endocarditis benefited from ^18^FDG PET–CT.

In patients with a suspected prosthetic valve endocarditis, both visual and quantitative assessments of ^18^FDG PET–CT have a high diagnostic sensitivity. To improve the diagnostic accuracy and interobserver reliability, a novel quantitative standardized cutoff of >2.0 for the ratio between ^18^FDG uptake around the affected valve and in the blood pool (standardized uptake value ratio) was proposed by Swart et al. [69]. This approach increased the sensitivity and specificity of ^18^FDG PET–CT to 100% and 90%, respectively.

**Figure 4:**
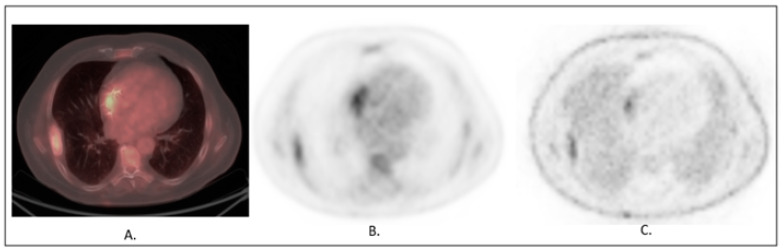
Infective Endocarditis in a patient with a cardiac device-related infective endocarditis. Axial scans at the level of the pacing leads: Infection focus in the right atrial leads. (**A**) Fused ^18^FDG PET and CT with tracer uptake in right atrium. (**B**) ^18^FDG PET attenuation-corrected scan. (**C**) ^18^FDG PET attenuation non-corrected scan showing uptake presence matching attenuation-corrected scan.

Several factors such as low disease activity (most frequently due to prolonged antimicrobial treatment), time after surgical intervention <3 months, prior use of surgical adhesives and physiological myocardial ^18^FDG accumulation have an adverse impact on the sensitivity [70], given that, to diminish the probability of false-negative ^18^FDG PET scans, it is essential to follow the procedural guidelines regarding high-fat, low-carbohydrate (HFLC) dietary preparation, administer heparin infusion if not contraindicated and adhere to a timely implementation of ^18^FDG PET–CT scanning [71]. It is of immense importance to utilize ^18^FDG PET–CT early in the diagnostic process, when the infection activity is high and the patient’s C-reactive protein levels are greater than 40 mg/L [69,72]. Prior use of surgical adhesives may result in a false-positive ^18^FDG uptake, which must be considered [69,73]. However, it should be noted that to avoid mistakes and to differentiate abnormal perivalvular uptake associated with IE from normal perivalvular uptake associated with a prosthetic valve, ^18^FDG PET–CT scans must be qualitatively interpreted by trained specialists, with obligatory analysis of attenuation-corrected and non-corrected images as recommended by societal guidelines [70] (Figure 4).

^18^FDG PET–CT plays a particularly important role in patients with IE associated with implantable devices (cardiac implanted device endocarditis—CDRIE). Performing PET imaging to visualize metabolic alterations before they become detectable on morphological studies is often crucial in deciding whether to explant the device [74,75]. Considering the unpredictable and often recalcitrant nature of IE, with cases not seldom complicated by heart failure and valvular structural destruction, this condition should be managed at a reference center by a dedicated endocarditis team that includes nuclear cardiologists.

## 7. Atherosclerotic Plaque Imaging

Beyond imaging infection, viability and myocardial perfusion, cardiac PET has the potential to depict the activity of the biology of atherosclerosis (Table 3).

We have learned by means of pathological studies that atherosclerosis is initiated by the deposition of cholesterol within the arterial intima, which is followed by an inflammatory response [76]. This leads to cell death and the formation of a large lipid-rich necrotic core which promotes further disease progression and eventually plaque rupture. Calcification occurs as a healing response to intense necrotic plaque inflammation. While the early stage of developing microcalcification is considered a common feature of ruptured or unstable plaques where healing is incomplete and inflammation remains active, macrocalcification leads to containment and stability of the plaque [77,78]. Over the past decade, PET was shown to enable non-invasive assessment of the aforementioned processes, which play a key role in plaque progression and rupture.

Multiple tracers depicting plaque inflammation and microcalcification have been evaluated. While both ^18^FDG and Somatostatin analogs (^68^GA-DOTATE) have shown hope in identifying inflamed atherosclerotic lesions, to date we lack outcome data that would demonstrate the prognostic value of these techniques. In contrast, ^18^F-sodium fluoride (^18^F-NaF)-, which has been traditionally used for imaging bone malignancies as it depicts areas of rapid bone-turnover, has shown great promise in imaging coronary atherosclerosis. The tracer diffuses via the capillary network into the extracellular fluid, and then it exchanges with hydroxyl groups on exposed regions of hydroxyapatite crystals on the calcification (bone) surface to form fluorapatite [79]. In cardiovascular ^18^F-NaF PET imaging, the surface area of hydroxyapatite appears to be the major factor affecting ^18^F-NaF uptake [80]. Indeed, ^18^F-NaF binding is highest in areas of microcalcification compared with large macroscopic deposits due to the very high surface area of hydroxyapatite in regions of powdery microcalcification [80].

Initial studies explored the utility of ^18^F-NaF PET in imaging recently ruptured plaques in patients with type one myocardial infarction [81]. Joshi et al. showed that intense ^1^⁸F-NaF uptake localizes to recent plaque rupture in patients with acute myocardial infarction. Moreover, in patients with stable coronary artery disease, ^1^⁸F-NaF uptake identified coronary plaques with high-risk features on intravascular ultrasound. The authors concluded that ^1^⁸F-NaF PET holds major promise as a tool for identifying high-risk and ruptured plaque, and potentially for informing the future management and treatment of patients with stable and unstable coronary artery disease [81]. These exciting results have led to further research which focused on exploring the clinical utility of the method and optimizing the technical aspects of coronary ^18^F-NaF PET. These latter efforts resulted in the development of dedicated tools for correcting for cardio-respiratory motion and optimized the acquisition, reconstruction and image analysis protocols. It was demonstrated that correcting for motion and the delay from tracer injection to image acquisition resulted in an improved reproducibility and image quality [82,83,84,85]. Dedicated reconstruction parameters along with a pancoronary uptake measure (the coronary microcalficiation activity) provide an opportunity for a patient-level assessment which is more closely associated with outcomes than single-pixel uptake values and has improved reproducibility [86,87,88,89,90]. Most recently, dedicated software and artificial intelligence tools have been shown to further streamline the analysis [91,92].

**Figure 5:**
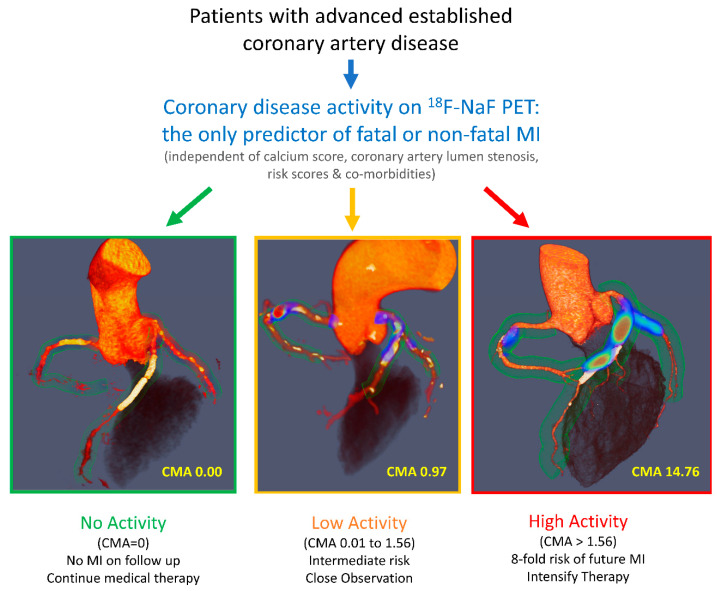
Imaging of atherosclerotic disease activity. ^18^F-sodium fluoride coronary PET imaging for imaging the coronary microcalcification activity (CMA) and risk stratification in patients with established coronary artery disease. Reprinted from JACC, Vol 75, Issue 24, Kwiecinski et al., Coronary 18F-sodium fluoride uptake predicts outcomes in patients with coronary artery disease, Pages 3061–3074, 2020, with permissions from Elsevier [98].

In parallel to these technical developments, observational studies have provided further multimodality and histological validation. ^18^F-NaF uptake has been linked to unfavorable plaque morphology on both invasive and non-invasive imaging [93,94,95]. Furthermore, it was demonstrated that ^18^F-NaF activity is associated with coronary inflammation measured on CT by means of assessments of the pericoronary adipose tissue attenuation [96,97]. Ultimately, by leveraging the technical refinements and the available observational data, a post hoc analysis showed that in patients with established coronary artery disease, ^18^F-NaF coronary uptake acts as a strong independent predictor of myocardial infarction (Figure 5) [98]. Importantly, ^18^F-NaF PET outperformed a wide range of established predictors including the presence of comorbidities; risk scores; coronary calcium scoring; and the presence, severity and extent of coronary artery disease. These exciting findings have been confirmed in further studies [99,100,101].

Recently, the utility of ^18^F-NaF PET beyond imaging coronary atherosclerosis has been extensively studied. As calcification is a hallmark of disease across a wide range of cardiovascular conditions, unsurprisingly, ^18^F-NaF uptake has been demonstrated to consistently predict progression and outcomes. This holds true for peripheral vascular atherosclerosis in relation to stent restenosis and ischemic stroke, aortic aneurysms in relation to aneurysm expansion requiring surgical repair and valvular heart disease [99,102,103,104,105]. The latter application includes both native aortic valve disease and bioprosthetic valve degeneration [106,107]. While, until recently, we lacked methods for the prediction of bioprosthesis failure, which can have catastrophic consequences unless detected and managed early, ^18^F-NaF lends itself to the population of patients with bioprosthetic valves. By identifying those who shall develop bioprosthesis failure, ^18^F-NaF provides hope that redo valve replacement, which is a high-risk undertaking, could be performed electively, thus mitigating the procedural risk.

Beyond the aforementioned radionuclides, currently multiple efforts have been made to develop novel tracers that would target key biological pathways involved in plaque progression and rupture. These include molecules targeting the endothelial vascular cell adhesion molecule-1 (VCAM-1) and the PFKFB3 enzyme, of which the expression is correlated with the presence of angiogenesis and vulnerable plaque formation [108,109]. Novel tracers have been also proposed for imaging infections. Instead of depicting areas of increased metabolism—which is the mechanism underlying ^18^FDG PET—new tracers target bacterial D-amino acids [110,111]. Beyond providing high specificity for bacterial infection, these tracers have the potential to clarify the etiology of infectious disease [111].

While over the past decades multiple non-invasive imaging approaches have been successfully adopted in several clinical applications, PET remains a key modality in modern practice. Despite the relatively high cost and ionizing radiation, due to its unique ability to image disease activity and metabolism at a molecular level, independence of acoustic windows (which hampers echocardiographic assessments) and utility in patients with implantable devices (which cannot undergo magnetic resonance imaging), cardiac PET imaging is a powerful non-invasive tool which can benefit patients at the point of care. Importantly, PET can be available in PET–CT or PET–MR configurations, with both hybrid scanners being advantageous in particular conditions. Although PET—CT is more widely employed, in diseases involving the myocardium the insights provided by MR imaging can be particularly valuable. Initially, a key limitation of PET/MR was the difficulty of obtaining attenuation correction—nowadays, this issue can be addressed with Dixon or Gradient Recalled Echo sequences [112]. While currently only a handful of radiotracers are widely utilized in the clinical setting, given the rapidly progressing development of new tracers, in the future we will likely witness growing uptake of this promising technology. In particular, in view of the progress in hardware with digital scanners which offer improved resolution, novel application of PET shall soon become adopted.

## Figures and Tables

**Table 1:** Characteristics of PET radionuclides.

Radionuclide	Half-Life	Source
^15^O	2 min	Cyclotron
^13^N	10 min	Cyclotron
^82^Rb	1.3 min	Generator
^18^F	110 min	Cyclotron
^68^Ga	68 min	Generator
^11^C	20 min	Cyclotron
^64^Cu	12.8 h	Cyclotron
^89^Zr	78 h	Cyclotron

**Table 2:** PET radiotracers for myocardial perfusion imaging.

Tracer	Isotope	Half-Life (min)	Production	First-Pass Extraction Fraction (%)	Uptake Mechanism	Availability	Stress Modality	MBF Quantification	Injected Activity (mCi)	Effective Dose (mSv)
^15^O-Water (H_2_^15^O)	^15^O	2	Cyclotron	~100	Free diffusion	+	Pharmacologic	yes	40	1.7
^13^N-ammonia (^13^NH_3_)	^13^N	9.96	Cyclotron	~80	Free diffusion and metabolic trapping (Na+/K+ ATPase)	+++	Exercise or pharmacologic With exercise, only static images available	with pharmacologic stress only	20	1.5
^82^Rubidium chloride (^82^RbCl)	^82^Rb	1.3	Generator	~65	Metabolic trapping (Na+/K+ ATPase)	++	Pharmacologic	yes	60	1.4

MBF—myocardial blood flow, availability graded +/++/+++.

**Table 3:** Current indications of PET in cardiovascular diseases and possible future applications.

Application	Tracer	Mechanism	Availability
Current PET Applications			
Myocardial perfusion	^15^O-Water (H_2_^15^O)	Free diffusion	+
	^13^N-ammonia (^13^NH_3_)	Free diffusion and metabolic trapping	+++
	^82^Rubidium chloride (^82^RbCl)	Metabolic trapping	++
	^18^F-flurpiridaz	Mitochondrial complex binding	Phase III clinical trial (NCT01347710)
Viability	^18^F-FDG	Glucose metabolism, GLUT4/GLUT1	+++
Inflammation, e.g., vasculitis	^18^F-FDG	Glucose metabolism Activated macropahes, neutrophils, CD4+leukocytes	+++
Infective endocarditis, implantable device infections	^18^F-FDG	Glucose metabolism Activated macrophages, neutrophils, CD4+leukocytes	+++
Cardiac malignancies	^18^F-FDG	Glucose metabolism Increased uptake by cancer cells	+++
Sarcoidosis	^18^F-FDG	Glucose metabolism Activated macrophages, neutrophils, CD4+leukocytes	+++
Future PET applications			
Sarcoidosis	^18^F-fluoromisonidazole (FMISO)	Tissue hypoxia	Research
Atherosclerosis imaging	^18^F-NaF	Active calcification, high-risk plaque	++ Research
Heart failure	^68^Ga or ^18^F-fibroblast activation protein inhibitor (FAPI)	Marker of activated fibroblasts Cardiac remodeling/injury	Research
Heart failure	^11^C-hydroxyephedrine (HED) [^18^F]flubrobenguane	Cardiac sympathetic innervation	Research
Atherosclerosis imaging	^68^Ga-DOTA-conjugated peptides: DOTATATE, DOTATOC, DOTANOC	SSTR2 High-risk lesions Activated macrophages—marker of atherosclerotic inflammation	Research
Atherosclerosis imaging	^64^Cu-DOTATATE		Research
Atherosclerosis imaging	^18^F-fluoromethylcholine	Activated macrophages—marker of atherosclerotic inflammation	Research
Atherosclerosis imaging	^68^Ga-Pentixafor	CXCR4	Research
Atherosclerosis imaging	^68^Ga–MacroP	Endothelial activation VCAM-1 Promising target for early atheroma detection	Research
Atherosclerosis imaging	NAMP–avidin–^68^Ga-BisDOTA	Endothelial activation VCAM-1 Promising target for early atheroma detection	Research
Atherosclerosis imaging	^18^F-fluorothymidine (FLT)	Cell proliferation, immune activation	Research
Cardiac amyloidosis	^18^F-florbetaben, ^18^F-florbetapir, ^18^F-flutemetamol,^11^C—Pittsburg Compound-B (PiB)	Amyloid deposits	Research

Availability graded +/++/+++.

## Data Availability

No new data were created or analyzed in this study. Data sharing is not applicable to this article.
